# Effects of Wonli Acupuncture Procedure in Patients with LSS: A Clinical, Retrospective Study

**DOI:** 10.1155/2014/212098

**Published:** 2014-06-22

**Authors:** Geon-Mok Lee, Eun-Yong Lee, Jong-Hyun Han, Kyong-Ha Cho, Se-Rin Kang, Sang-Hoon Yoon

**Affiliations:** ^1^Lee-Geonmok Wonli Korean Medicine Hospital, 196 Dongjak-daero, Seocho-gu, Seoul 137-829, Republic of Korea; ^2^Department of Acupuncture and Moxibustion, College of Korean Medicine, Semyung University, 65 Semyeong-ro, Jecheon-si 390-711, Republic of Korea; ^3^Department of Pharmacology, College of Korean Medicine, Wonkwang University, 460 Iksan-daero, Iksan-si 570-749, Republic of Korea

## Abstract

*Background*. Lumbar spinal stenosis (LSS) is a disease with increasing prevalence due to prolongation of average life span. Despite various treatment methods, many limitations remain unsolved. *Objective*. We are reporting cases of patients who have been treated with Wonli Acupuncture, a method of treating LSS by directly approaching the intervertebral foramen and interlaminar space with acupuncture needles different from those used in original acupuncture. *Methods*. A total of 82 patients with LSS were treated with Wonli Acupuncture, and out of those, 47 patients without exclusion criteria were selected for the following research. We compared the pretreatment VAS and ODI scores based on 1-year follow-up measurements. *Results*. The ODI value dropped by 15.3 ± 24.8 on average (from 35.2 ± 19.9 at the baseline to 19.8 ± 20.6 at the reading) (*P* < 0.01) and the average VAS also dropped by 19.2 ± 37.2 (from 60.7 ± 23.1 at baseline to 41.5 ± 31.9 at the reading) (*P* < 0.01). *Conclusions*. Wonli Acupuncture was found to have clinical efficacy for lumbar spinal stenosis.

## 1. Introduction

Spinal stenosis is defined as the narrowing of spinal canal due to the surrounding bone and soft tissues which results in physical pressure on the nerve root [1]. This is a disease common for the aged, generating lumbago and peripheral neuralgia as well as dysbasia and severe disability [2, 3]. As a result, the lumbar spinal stenosis is the main reason for spine surgery for people older than 65 [4–6]. However, the effect of surgical therapy is limited only to spine decompression, nerve root decompression, and lumbar body fusion of regressive lumbar spondylosis [7]. In addition, the nonoperative therapies such as pharmacological therapy, physical therapy, and manipulation have also little therapeutic effect and have weak clinical basis of use [8].

The lumbar epidural steroid injection is frequently used for patients with lumbar spinal stenosis [9–11]. But this therapy often fails to improve melosalgia and claudication [12, 13]. This is because spinal stenosis is generated by the complicated pathological process of fibrosis around the space-occupying lesions or nerves, causing interference of blood flow, ischemia, venostasis, venal fibrosis, and, in turn, nutrition deficiency [14–17].

To address these limitations, without using existing therapeutic modalities, a method of using physical tools was developed to remove attached tissues, to reduce pressure on the nerve, and to improve surrounding blood circulation. This is acupotomy. Developed by Zhou Han Zhang of China, acupotomy is advantageous for incision and sublation of attached tissues with its thick needle body and its end resembling a sharp knife [18]. Acupotomy has been used for spinal stenosis therapy and clinical studies have been published [19, 20].

However, acupotomy can damage tissues in the spinal canal such as nerve and blood vessels due to its sharp end. Therefore, we used a rounded tip with more curvature than original tools of acupotomy. We named this Wonli Acupuncture from one of the nine ancient classical needles. To date, there have been no studies revealing the therapeutic effect of Wonli Acupuncture needle on spinal stenosis. Accordingly, this case study research was designed to reveal the effect of the Wonli Acupuncture procedure on direct exfoliation of synechia around nerves.

## 2. Methods and Material

### 2.1. Subjects

This retrospective case study was conducted at Seoul Wonkwang Korean Medicine Hospital, Seoul, Korea. The study protocol was approved by the institutional review board (IRB) of Semyung University Hospital (Chungju, Korea) with an assigned number of 1402-01. We followed guidelines of Standards for Reporting Interventions in Controlled Trials of Acupuncture (STRICTA); see Table 1. As a retrospective case study, written informed consent was not required. Between November 2012 and January 2013, we investigated 47 patients to evaluate the clinical efficacy of Wonli Acupuncture procedure on lumbar spinal spenosis (LSS); see Table 2.

### 2.2. Inclusion and Exclusion Criteria

#### 2.2.1. Inclusion Criteria

- Not relieved for at least 3 months by conservative treatments (exercise, analgesics, and epidural steroid injection);
- gluteal pain;
- radiating pain;
- provocative factors:
  - neurogenic claudication induced by walking or positional change;
- palliative factors:
  - symptomatic relief with forward flexion, sitting, and recumbence.

#### 2.2.2. Exclusion Criteria

- Coagulopathy;
- bleeding diathesis;
- abnormal findings by ECG, blood test;
- acute back and leg pain;
- history of prior spine surgery;
- abnormal tendon reflexes;
- muscle atrophy;
- spondylolysis and spondylolisthesis;
- degenerative scoliosis;
- medical conditions that can affect radicular pain, that is, Lou Gehrig's disease and Parkinson's disease.

### 2.3. Diagnostic Criteria

The diagnosis of patients was based on clinical and radiological findings. Despite clinical definition that relies on anatomical findings, severity of LSS depends primarily on the patient's description of their symptoms and physical examination. Studies have reported a high mismatch between symptoms and radiological findings. Hence, when interventions are considered, correlation between symptoms and radiological findings is required [22–24]. Studies indicate MRI as the most appropriate diagnostic method to confirm the presence of anatomic deformation for surgical intervention [25]. In order to coordinate the level of treatment we evaluated whether the patient's radiological findings corresponded with described symptoms and physical findings. Once the specific lumbar joint with anatomical deformation via MRI findings corresponded with symptoms, it was considered as target for treatment.

### 2.4. Description of Intervention

A doctor of Korean medicine with 19 years of clinical experience led all procedures while patients received Wonli Acupuncture in an aseptic environment. The pre-/postacupuncture effect was investigated by a different doctor of Korean medicine with 3 years of clinical experience, who did not participate in the procedure.

### 2.5. Assessment

Two outcome measures were selected to evaluate the effectiveness of the treatment: visual analogue score (VAS) and Oswestry disability index (ODI), both measured before and after treatment; see Table 3 and Figure 9. The duration from procedure to measurement was approximately one year. The VAS is a horizontal line, 100 mm in length, anchored by word descriptors at each end. The patient marks on the line the point that they feel best represents their perception of their current state. The VAS score is determined by measuring in millimeters from the left end to the point that the patient marks. ODI is the principal condition-specific outcomes measure used in the management of spinal disorders [26]. The patient questionnaire is composed of topics such as intensity of pain, ability to walk, sit, and stand, sexual function, social life, and sleep quality. Each question is scored on a scale of 0–5 with the first statement being zero and indicating the least amount of disability and the last statement of 5 indicating most severe disability. The index is scored from 0 to 100. Zero is equated with no disability and 100 being maximum disability [26].

### 2.6. Technique of Wonli Acupuncture Procedure

#### 2.6.1. Posture

The patients were instructed to lie on a table in the prone position

#### 2.6.2. Approach Point

For a safe approach, the anatomic point and needle points are marked on the patient's skin with a marker.

**Figure 1:**
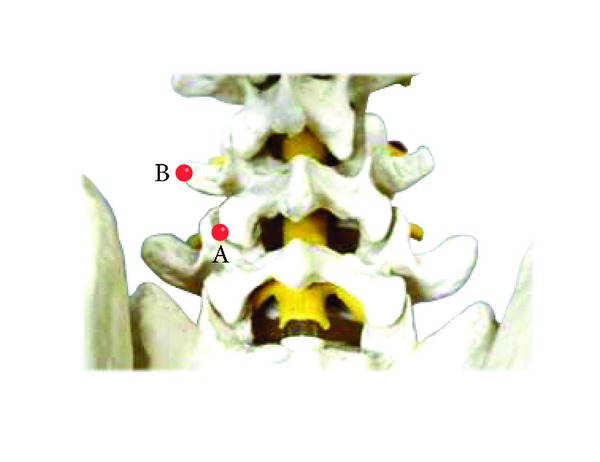
Approach points A and B.

Figure 1, point A (lumbar zygapophyseal joint): by correlating MRI/X-ray image findings with patients symptoms, the lumbar disc level for therapy is selected. The acupuncture target point is the zygapophyseal joint at the corresponding lumbar disc level. The skin over the corresponding zygapophyseal joint is marked. The zygapophyseal joint is positioned at 12~15 mm on both sides of the lumbar interspinous point with some patient to patient variability.

Figure 1, point B (tip of transverse process): the acupuncture target point is the intervertebral foramen at the lumbar disk level where the lesion is suspected. The acupuncture entrance point is the tip of the transverse process of the lumbar at a higher disk level than the level of suspected lesion. This is 4 cm away from the interspinous point.

#### 2.6.3. Creating an Aseptic Operating Environment

The skin over the surgical field is prepped with povidone iodide antiseptic solution and alcohol solution twice and covered with a germ-free fabric to secure antiseptic environment. The operator wears surgical gloves, mask, and operating gown to maintain aseptic environment throughout the procedure.

#### 2.6.4. Therapeutic Tool

**Figure 2:**
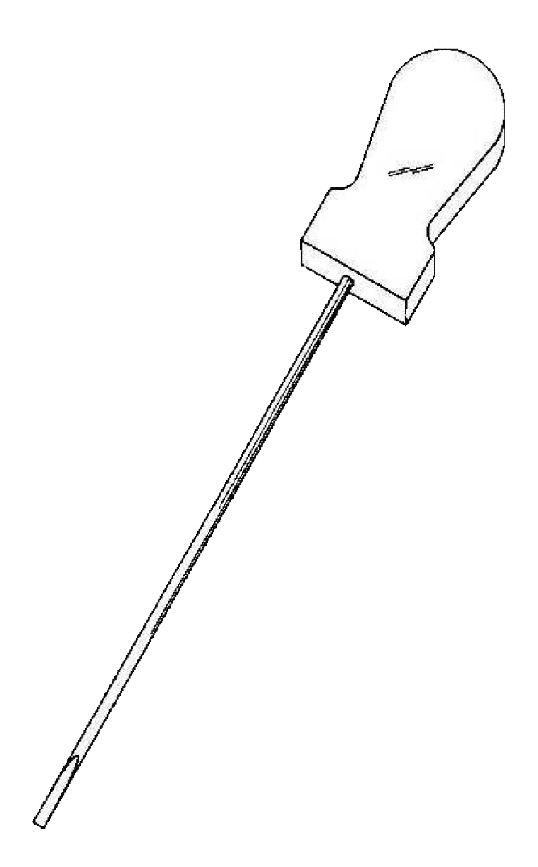
Acupuncture needle 1.

Acupuncture needle 1: “Dongbang Dochim,” made by Dongbang acupuncture needle company, is 1 mm in diameter, 105 mm in overall length, 80 mm in length of needle, and 25 mm in length of handle and with a flat blade of 5 mm at the tip (see Figure 2).

**Figure 3:**
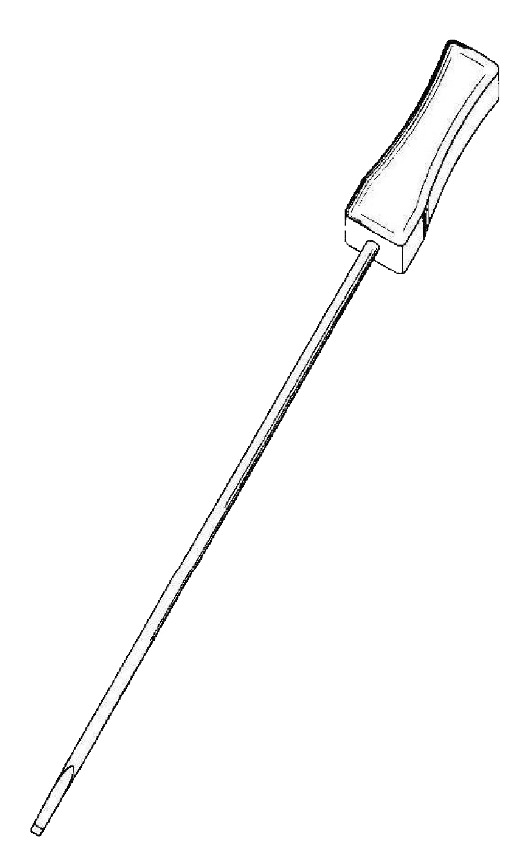
Acupuncture needle 2.

Acupuncture needle 2: It is 2.5 mm in diameter, 145 mm in overall length, 115 mm in length of needle, and 30 mm in length of handle. The tip is a flat blade 11 mm in length (see Figure 3).

**Figure 4:**
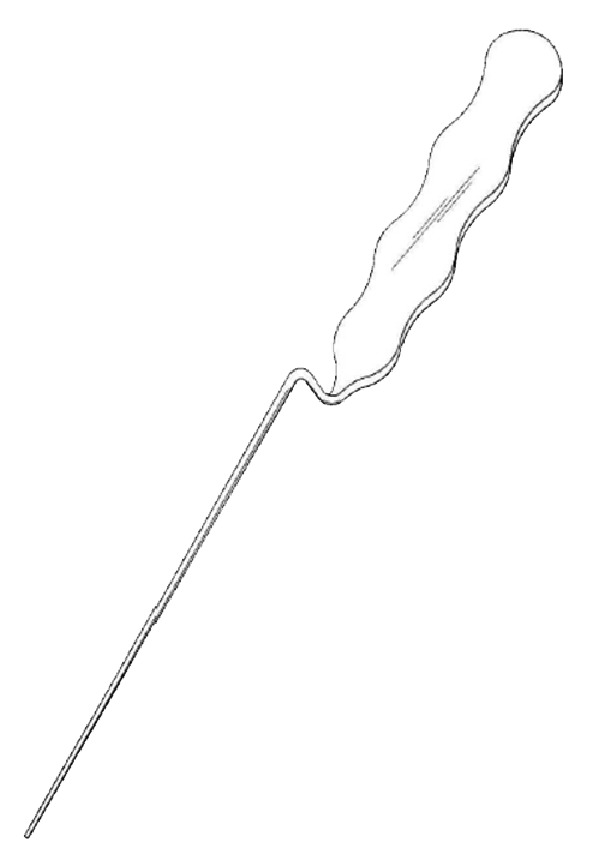
Acupuncture needle 3.

Acupuncture needle 3: it is a Z-shaped acupuncture needle, 2 mm in diameter, 255 mm in overall length, 150 mm in length of needle, and 105 mm in length of handle, with a blunt and round tip (see Figure 4).

**Figure 5:**
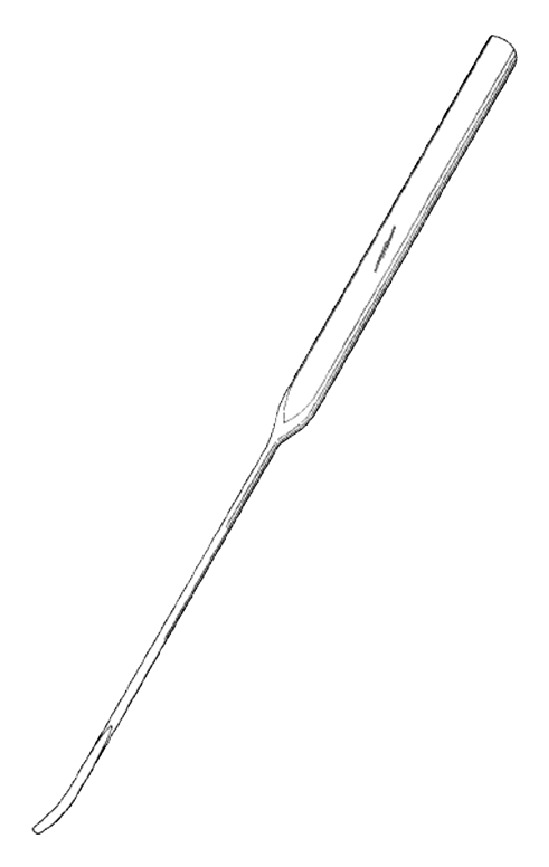
Acupuncture needle 4.

Acupuncture needle 4: it is 2 mm in diameter, 170 mm in overall length, 85 mm in length of needle, and 85 mm in length of handle. 15 mm of the needle tip is streamlined and bent at an approximately 20° angle, with blunt and round tip (see Figure 5).

#### 2.6.5. Operation Method

**Figure 6:**
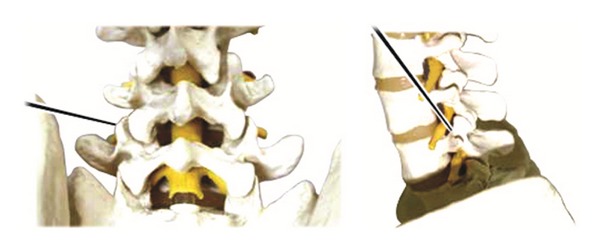
Acupoint of acupuncture needle 3.

**Figure 7:**
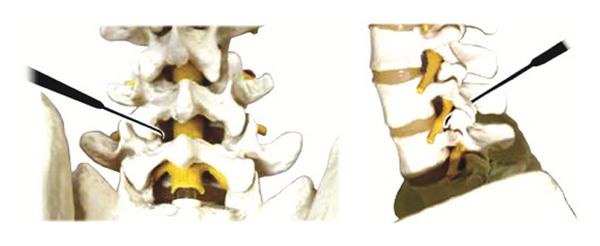
Acupoint of acupuncture needle 4.

1. Insert acupuncture needle 1 through point A perpendicular to the skin toward the lumbar zygapophyseal joint. Keep the blade parallel with the articular surface of facet joint and incise the facet joint capsule using acupuncture needle 1. Incise the solidification in/out/up/down of the articular process along the bone surface and then incise the synechia of multifidus muscles and rotator muscles in parallel with the muscle grain direction to stretch the shortened muscle.
2. Use acupuncture needle 2 to penetrate through the epidermis at points A and B to secure the inserting point of acupuncture needles 3 and 4. Incise the epidermis layer only to the depth of 10~15 mm.
3. Holding the handle of acupuncture needle 3 insert it perpendicular to point B to a depth of 10 mm, passing through the epidermis. Then, keeping the angle with the epidermis at 45 degrees, insert the needle vertically to the depth of 8~12 cm toward the intervertebral foramen of the disk level until it reaches the anterior part of superior articular process. After reaching the intervertebral foramen of the spine, exfoliate the synechia of soft tissues in the vicinity of the intervertebral foramen located on the anterior side of the facet joint and inside the pedicle, all along the bone surface (see Figure 6).
4. Insert acupuncture needle 4 perpendicular to point A until it reaches the facet joint and then slowly insert it along the internal part of the surface of facet joint bone. Incise the adhered and thickened ligamentum flavum in the interlaminar space along the bone surface. Repeat this procedure until resistance of the yellow ligaments is resolved, indicated by loose feeling. When the yellow ligaments are exfoliated sufficiently, move further toward the intervertebral foramen to exfoliate the synechia in the spinal canal (see Figure 7).

## 3. Results

### 3.1. Study Population

**Figure 8:**
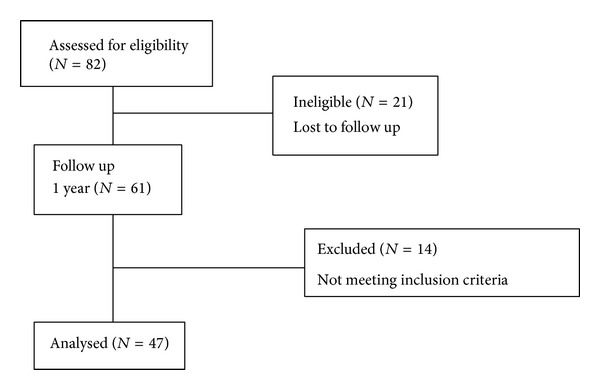
Flow chart.

**Figure 9:**
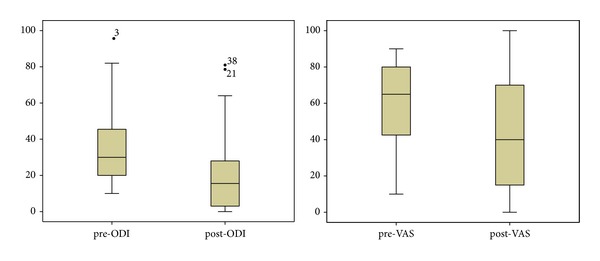
Coefficient of quartile deviation of pre- and post-ODI; pre- and post-VAS.

During the research period, the number of stenosis patients of whom we measured ODI and VAS was 82. After a year, we received a total of 61 responses. Among the 61 responders, 14 were excluded. One had Lou Gehrig's disease and others had other spinal deformities such as anterolisthesis, retrusion, and serious scoliosis. A total of 47 stenosis patients met our research criteria; see Figure 8.

The average age of patients was 57.9 ± 10.0 (58.1 ± 10.0 for males and 57.7 ± 10.1 for females). The males numbered 20 (42.6%) and the females numbered 27 (57.4%). The average pain period before therapy was 38.8 ± 77.4 months (29.9 ± 66.9 months for males and 45.4 ± 86.0 months for females). Regarding the affected spinal level, L4-L5 was affected for 45 patients (95.7%), L3-L4 27 patients (57.4%), L5-S1 26 (55.3%), L2-L3 15 (31.9%), and L1-L2 4 (8.5%). Nineteen patients (40.4%) had a problem with both segments of the spine, 12 patients (25.5%) with 3 segments, 8 patients (17.0%) with a segment, and 3 patients (6.4%) with 5 segments. Regarding the pain aspect of the patients, 39 patients (83%) had unilateral radiating pain, 6 patients (12.8%) had bilateral radiating pain, and 2 patients (4.3%) had no radiating pain but complained only of lumbago.

Twenty-one patients (44.7%) received the Wonli Acupuncture procedure once, 22 patients (46.8%) twice, 3 patients (6.4%) 3 times, and one patient (2.1%) 4 times. No patient suffered from serious or life threatening complications or loss of physical function after the procedure.

### 3.2. Outcomes

Setting 100 points as full marks for the patients following the Wonli Acupuncture procedure the ODI value dropped by 15.3 ± 24.8 on average (from 35.2 ± 19.9 baseline to 19.8 ± 20.6 at the reading) (*P* < 0.01) and the average VAS also dropped by 19.2 ± 37.2 (from 60.7 ± 23.1 at baseline to 41.5 ± 31.9 at the reading) (*P* < 0.01).

## 4. Discussion

When we compared ODI and VAS of the patients before and after therapy, the Wonli Acupuncture procedure was found to have clinical efficacy for lumbar spinal stenosis. The Wonli Acupuncture procedure was developed by Dr. Lee Geon Mok by improving the existing Chinese acupotomy tools and technology.

Acupotomy is an acupuncture procedure developed for synechia, nodules, and cicatrix caused by soft tissue damage, in short, a combination of acupuncture and ectomy. Dr. Zhou Han Zhang of China tried this method for the first time by using a therapeutic tool called the small acupotomy, integrating a needle and a knife [18]. Acupotomy is a therapy to exfoliate synechia by applying a knife shaped needle tip to the synechia around muscles and ligaments [27]. Once recovered by acupotomy, the synechia is removed and tissues are free to move during activity. The area functions normally and the pain is resolved due to smooth interaction [28]. In China, acupotomy is already used for stenosis and related clinical researches have been published [19, 20].

However, acupuncture needle 1, most commonly used in China, has a knife at the needle tip. And despite minimizing the damage during insertion, there are limitations in directly entering the spinal canal and manipulating the needle due to the risk of nerve and blood vessel damage. Wonli Acupuncture procedure utilizes needle 1 only in the area surrounding the facet joint and then utilizes acupuncture needles 3 and 4, whose needle tips are round, in the spinal canal. Acupuncture needle 3 and needle 4 are both called the Wonli Acupuncture. These needles are shaped with higher density to avoid damaging nerves and blood vessels, enabling them to enter the spinal canal with higher safety level compared to acupuncture needle 1. In particular, acupuncture needle 4's tip is curved in order to approach the spinal canal along the lamina.

Moreover, existing acupotomy therapy was not satisfactory in bringing explicit and fast relief to patients due to the large areas needing to be manipulated multiple times. Dr. Lee devised this new acupuncture procedure to reduce the number of treatments by increasing the effectiveness and safety. Dr. Lee named this series of treatment process based on Wonli Acupuncture, the “Wonli Acupuncture procedure.”

Step 1 is the process of resolving synechia of articular process and facet joint capsules using acupuncture needle 1. In this step, to treat arthritis, the deformed articular process hyperplasia and joint capsule hypertrophy are exfoliated. In addition, when the facet joint and motor units of vertebral body are under stress, the balance is lost and displacement of the intervertebral joint occurs [29]. This, in turn, affects the spinal canal and lateral recess structure [30]. This method of exfoliating the area between facet joints improves the mobility of the shifted joints and hence recovers the right position.

Acupuncture needle 3 is a tool resolving the synechia of intervertebral foramen. When lumbar spinal stenosis occurs, the tissues of intervertebral foramen (periosteum, connective tissue, and nerve root adventitia) tend to harden and thicken, often causing calcification [29]. Exfoliation of the adhesion of intervertebral foramen along the bone surface with acupuncture needle 3, the synechia of nerves, blood vessels, and membranes are mechanically separated. When perineural adhesion is resolved, nerves become more active. As reports of intervertebral foramen decompression, exfoliating the intervertebral foramen extends the nerve root length by 5~8 mm and increases the creep rate [31]. This method is also useful for separating the synechia around the nerve, improving the blood circulation, and resolving its avascular inflammation, which is believed to improve the intermittent claudication.

Thickened yellow ligaments can be removed from the vertical lamina by inserting acupuncture needle 4 into the interlaminar space. Once exfoliated, the resistance of the yellow ligaments is hardly felt. When the ligaments are satisfactorily exfoliated, moving the needle from the lamina to the deep inside region of intervertebral foramen exfoliates the spinal canal. With a snap, the conglutinated soft tissue of the nerve and disk is taken off the spinal canal.

Likewise, venous congestion can be reduced by securing the spinal canal space. Venous congestion may cause circulatory disturbance and further claudication [32]. When this is improved, microcirculation also improves. Thus, the blood circulates more smoothly towards the nerve and chronic neuritis is relieved, generating a pain relieving chain reaction. As a result, central stenosis and lateral recess stenosis are solved simultaneously.

When we compared the radiological images of pre- and post-Wonli Acupuncture procedure, we observed increases in the distance between the interbodies in several cases. We collected 26 postprocedure X-ray images of 47 patients with LSS in L4-5. The improvement of distance between L4 and L5 was 6.60 mm ± 2.98 to 7.27 ± 2.78 mm, with an average increase of 0.67 mm (*P* < 0.05). Based on these results further researches are expected in the future.

Stenosis is a result of cumulative damage and consequent pathological changes such as synechia, cicatrix, and contractions. Inevitably, these changes affect the motor function of body's motor unit [33]. When lesions are exfoliated, the pressure on intervertebral motor unit is effectively removed and the distance between interbodies increases, leading to reduction of the pressure on nerves.

In conclusion, we believe the Wonli Acupuncture procedure is a series of processes, not only widening the spinal canal physically but also inducing a chain reaction through exfoliation. When the Wonli Acupuncture procedure exfoliates the synechia, venous congestion is improved and microcirculation becomes active, resolving the edema in the spinal canal. Last but not least, both the spinal canal and the epidural space expand physically and chemically, reducing the pressure on nerves and improving the symptoms.

When we observed the therapy results, some patients got better right after a single procedure while some improved gradually over several months. In the latter case, they seemed to take time recovering from denaturalization of the damaged nerve and its surrounding tissues. Not only this, but according to clinical observation patients who obtained no effect from the Wonli Acupuncture procedure seemed to have experienced regressive changes of the spine. Judging from this, they may have gotten worse despite their recovery in the first postprocedure period.

Some people obtained tangible effect from a single Wonli Acupuncture procedure session while some underwent two or more sessions until they received tangible effect. For those who had light synechia, one session was enough to separate the synechia from the nerve. However, for those who had serious synechia and thick yellow ligaments, patients improved at first and then aggravated again or got even worse without any improvement after the first session. This is thought to have been caused by readhesion, despite the existing synechia being exfoliated. We assume regular exfoliation is necessary for preventing recurrence of synechia. Therefore, repeating Wonli Acupuncture procedure is necessary.

Modern medicine generally offers surgery and epidural steroid injection for lumbar spinal stenosis [34]. Surgical treatment is good for relatively faster recovery but it has been reported to have a side effect of spine instability as it removes the spine to widen the space [35]. The failure rate of spine surgery is known to be as high as 10~40% [36]. In addition, based on the anti-inflammatory effect of steroids, the epidural steroid injection has been reported to be effective in the beginning but symptoms recur as time passes [37]. Currently, a method was introduced in Korea which uses a transforaminal balloon to treat lateral foraminal stenosis. According to the report, 18.8% of the patients were relieved of more than 50% of their pain for 52 weeks [38].

Wonli Acupuncture procedure is a method of spinal canal therapy using acupuncture as little as possible with no reported allergy or side effects to date. We must note that this procedure can be applied over and over again whenever the symptom reoccurs and it is applicable not only to lateral foraminal stenosis but also to central, lateral recess, foraminal, and extraforaminal region.

## 5. Limitations

This study has the inherent limitations of a case series and retrospective research. In the future, additional studies are required, including more diversified sample sizes, longer prospective randomized clinical trials, and comparison between Wonli Acupuncture procedure and other stenosis therapies.

The current research was based on the comparison between ODI and VAS scores, excluding muscular atrophy or paralysis and the patients with history of prior surgery. Patients with muscle atrophy, unlike those with simple radiating pain, would show various therapeutic outcomes and their objective estimation would be difficult. Patients who had surgery on the lumbar spine prior to our procedure with possibility of more complicated iatrogenic stricture were excluded for the homogeneity of the patient group. In view of the mechanism, this therapy is thought to be applicable to the patient with past surgery history and therefore these patient groups should be further studied in the future.

## Supplementary Material

Wonli Acupuncture Procedure in Patient with LSS. A movie demonstrating the technique. 1) Desquamate the ligaments around facet joint with 1mm thick Acupuncture Needle 1. 2) Make an opening enough for procedure, using Acupuncture Needle 2. 3) Using Acupuncture Needle 3, desquamate the soft tissues conglutinant around the intervertebral foramen. 4) Through interlaminar space, insert the Acupuncture Needle 4 and desquamate the conglutinant and thickened ligamentum flavum.

## Figures and Tables

**Table 1:** Therapy by the STRICTA recommendation [21].

Item	Details
(1) Acupuncture rationale	(1a) Style of acupuncture: Wonli Acupuncture procedure
	(1b) Reasoning for therapy provided, based on historical context, literature sources, and/or consensus methods, with appropriate references: we incised the conglutinated part from the fiberized lesion to promote injury recovery, tissue proliferation, and fortification [18]
	(1c) Extent to which therapy was varied: each patient received acupuncture independently according to his/her symptoms. On the basis of imaging studies and patient symptoms, we determined each spinal disk level and treated points A and B. The number of the therapy points was dependent on the disk level.

(2) Details of needling	(2a) Number of acupuncture needle insertions per subject per session (mean and range where relevant): 4 to 12 per session
	(2b) Names (or location if no standard name) of points used (uni/bilateral): zygapophyseal joint (both sides), the tip of the transverse process (both sides)
	(2c) Depth of insertion, based on a specified unit of measurement or on a particular tissue level: 4~8 cm deep (acupuncture for anatomical structure)
	(2d) Response sought: pain and twinge (nerve stimulation)
	(2e) Needle stimulation: manual
	(2f) Needle retention time: postprocedure removal
	(2g) Needle type: (i) acupuncture needle 1: “Dongbang Dochim,” made by Dongbang acupuncture needle company, 1 mm in diameter, 105 mm in overall length, 80 mm in length of needle, and 25 mm in length of handle and with a flat blade of 5 mm at the tip (ii) acupuncture needle 2: 2.5 mm in diameter, 145 mm in overall length, 115 mm in length of needle, and 30 mm in length of handle; the tip is a flat blade 11 mm in length (iii) acupuncture needle 3: a Z-shaped acupuncture needle, 2 mm in diameter, 255 mm in overall length, 150 mm in length of needle, and 105 mm in length of handle, with a blunt and round tip (iv) acupuncture needle 4: 2 mm in diameter, 170 mm in overall length, 85 mm in length of needle, and 85 mm in length of handle. 15 mm of the needle tip is streamlined and bent at an approximately 20° angle, with blunt and round tip.

(3) Therapy regimen	(3a) Number of therapy sessions: 1 to 4 sessions
	(3b) Frequency and duration of therapy sessions: once (or more for patients who report continued pain).

(4) Other components of therapy	(4a) Details of other interventions administered to the acupuncture group: after Wonli Acupuncture procedure all patients were admitted for 3 days of observation: all patients were administered extracts of Eungyosan 600 mg and Baenongsan 500 mg as herbal anti-inflammatory drugs (3 times a day for 3 days); Eungyosan, made by Kihwabio pharmaceutical company, consists of 10 ingredients, namely, Lonicera japonica Thunberg 710 mg, Forsythia koreana Nakai 710 mg, Mentha arvensis Linne var. piperascens Malinvaud 426 mg, Platycodon grandiflorum (Jacq) Nakai 426 mg, Glycyrrhiza uralensis Fisch 426 mg, Glycine max Merrill 356 mg, Arctii Fructus 356 mg, Lophatherum gracile 284 mg, Schizonepeta tenuifolia var. japonica (Maxim.) Kitag. 284 mg, and Saiga Tataricae Cornu 22 mg; Baenongsan, made by GP pharmaceutical company, consists of 6 ingredients, namely, Citrus aurantium L. 500 mg, Platycodon grandiflorum (Jacq) Nakai 670 mg, Paeonia lactiflora Pall. 500 mg, Zingiber officinale Roscoe 170 mg, Zizyphus jujuba var. inermis 500 mg, and Glycyrrhiza uralensis 500 mg
	(4b) Setting and context of therapy, including instructions to practitioners and information and explanations to patients: collection of data before and after the treatment was carried out by the doctor of Korean medicine who made the diagnosis; the treatment was done by a different doctor of Korean medicine in a separate space, on the basis of patient's medical record and imaging diagnosis.

(5) Practitioner background	(5) Description of participating acupuncturists (qualification or professional affiliation, years in acupuncture practice, and other relevant experience): (i) qualification: acupuncture specialist (ii) membership: Chairman of Korean Medical Institute of Acupotomy, Regular Member of the Korean Acupuncture & Moxibustion Society (iii) relevant training period: 5 years (iv) clinical experience: 19 years (v) professional technology for a certain disease: developed Wonli Acupuncture procedure.

(6) Control or comparison interventions	(6a) Rationale for control or comparison intervention in the context of research question, with sources that justify this choice: no comparison group
	(6b) Precise description of the control or comparison interventions; if sham acupuncture or any other type of acupuncture-like control is used, provide details as for items 1 to 3 above: no comparison group.

**Table 2:** Subject demographics.

Patients (*N*)	47
Age (years)	57.9 ± 10.0
Gender (M/F)	20/27
Duration of symptom (months)	45.4 ± 86.0
Oswestry disability index (%)	35.2 ± 19.9
Visual analogue scale	60.7 ± 23.1

Involved level	
L4-5 (*n*)	45 (95.7%)
L3-4 (*n*)	27 (57.4%)
L5-S1 (*n*)	26 (55.3%)

Radicular pain	
Unilateral (*n*)	39 (83%)
Bilateral	6 (12.8%)
Only back pain	2 (4.4%)

**Table 3:** Change of VAS and ODI scale of 1 year after intervension.

	Baseline	Follow-up (1 year)
ODI	35.2 ± 19.9	19.8 ± 20.6∗∗
VAS	60.7 ± 23.1	41.5 ± 31.9∗∗
>50% improvement ODI		24/47 (51.1%)
>50% improvement VAS		18/47 (38.3%)

**P < 0.01.
